# Minimum meal frequency and associated factors among children aged 6–23 months in Sub-Saharan Africa: a multilevel analysis of the demographic and health survey data

**DOI:** 10.3389/fpubh.2024.1468701

**Published:** 2024-11-22

**Authors:** Tsion Mulat Tebeje, Mesfin Abebe, Solomon Hailemariam Tesfaye, Binyam Tariku Seboka, Girum Shibeshi Argaw, Beminate Lemma Seifu, Kusse Urmale Mare, Fantu Mamo Aragaw

**Affiliations:** ^1^School of Public Health, College of Health Science and Medicine, Dilla University, Dilla, Ethiopia; ^2^Department of Midwifery, College of Health Science and Medicine, Dilla University, Dilla, Ethiopia; ^3^Department of Public Health, College of Medicine and Health Sciences, Jigjiga University, Jijiga, Ethiopia; ^4^Department of Public Health, College of Medicine and Health Science, Samara University, Semera, Ethiopia; ^5^Department of Nursing, College of Medicine and Health Sciences, Samara University, Semera, Ethiopia; ^6^Department of Epidemiology and Biostatistics, College of Medicine and Health Sciences, Institute of Public Health, University of Gondar, Gondar, Ethiopia

**Keywords:** minimum meal frequency, Sub-Saharan Africa, DHS, multilevel analysis, children

## Abstract

**Background:**

Worldwide, approximately half of all children are not provided the minimum meal frequency (MMF). Sub-Saharan Africa (SSA) had the lowest proportion of children aged 6–23 months who met the requirements of the complementary feeding indicators, including MMF. Ensuring adequate meal frequency poses a challenge in numerous developing countries, particularly in regions characterized by low household food security, such as SSA. Therefore, this study aimed to assess the pooled prevalence of MMF and its associated factors in SSA via the most recent demographic and health survey data.

**Methods:**

A total of 100,526 weighted samples from demographic and health survey datasets of 35 SSA countries were used. A multilevel Poisson regression model with robust variance was applied to identify factors associated with MMF, and the model with the lowest deviance was the best-fitted model. An adjusted prevalence ratio with a 95% confidence interval (CI) was reported, and variables with a *p* < 0.05 were considered statistically significant.

**Results:**

The pooled prevalence of MMF among children aged 6–23 months in SSA was 38.47% (95% CI: 34.97–41.97), which ranged from 21.41% in Liberia to 63.98% in Madagascar. According to the subgroup analysis, the pooled magnitude of MMF in central, west, east, and southern Africa was 36.42, 35.46, 39.97, and 50.69%, respectively. Marital status, maternal education level, sex of household head, working status, wealth index, media exposure, age of the child, postnatal check-up, breastfeeding status, residence, and SSA regions were significantly associated with minimum meal frequency.

**Conclusion:**

Less than forty percent of infants and young children in SSA receive the minimum recommended meal frequency, which is relatively low. This presents a notable difficulty in efforts to prevent malnutrition and attain sustainable development goals related to health and nutrition on the continent. Therefore, priority should be given to empowering women, promoting breastfeeding and postnatal check-ups, targeting infants who just started complementary feeding, and spreading information through media.

## Introduction

Undernutrition is a contributing cause of child morbidity and mortality worldwide (1, 2). Adequate nutrition is vital for the proper health and development of children (3). The age range of 6–23 months presents a critical window to address childhood malnutrition as it is a period marked by growth faltering and increased nutritional needs that necessitate energy-dense and nutrient-rich foods (4). The nutritional value of food is paramount to the health and wellbeing of families, especially children who need essential nutrients to help them grow, develop, and reach their full physical and cognitive potential (5).

Minimum meal frequency (MMF), one of the complementary feeding indicators, is utilized as a proxy indicator for evaluating a child’s energy requirements and emphasizes the number of times that children of different ages are offered meals other than breast milk and breastfeeding (6, 7). Non-breastfed children should be given four or five meals throughout the day, incorporating both milk feeds and solid or semisolid feeds and allowing for one to two snacks on the basis of their individual preferences (8–10).

Enhancing infant and young child feeding (IYCF) practices is recognized as a critical component in enhancing child survival rates and fostering optimal growth and development (11–13). Inadequate infant and young child feeding practices during the first 2 years of life have detrimental consequences, including irreversible stunting, impaired cognitive development, and a substantial increase in the risk of both chronic and infectious diseases (14).

Ensuring appropriate complementary feeding practices is crucial for enabling the growth and development of children (15). Suboptimal complementary feeding practices in infants and children can increase the likelihood of undernutrition, illness, and mortality (16). The likelihood of malnutrition in infants and young children increases significantly after the age of 6 months (4). Common childhood illnesses such as diarrhea and infections, along with high nutritional requirements beyond breast milk, are crucial for maintaining a child’s normal development (4).

To achieve one of the targets of goal two of the sustainable development goal (SDG), which is ending all forms of malnutrition, complementary feeding practices, including MMF, should be appropriate, as they are the major determinants of malnutrition (17). The presence of nutritional deficiencies in the first 2 years of life can lead to impaired cognitive development, compromised educational outcomes, and reduced economic productivity (18, 19). Children suffering from malnutrition are more prone to illness and endure the lifelong consequences of inadequate nutrition, which may have intergenerational effects (18, 20).

In numerous countries worldwide, fewer than one-fourth of infants aged 6–23 months fulfill the criteria for dietary diversity and feeding frequency, resulting in only a few children being provided nutritionally adequate and diverse foods (21). Worldwide, about half of all children are not provided MMF, and even in the wealthiest households in regions such as West and Central Africa, Eastern and Southern Africa, and South Asia, the proportion of children who consume the recommended minimum meals per day is quite low (22). According to a publication on 49 national surveys from low-and middle-income countries on dietary quality, SSA had the lowest proportion of children aged 6–23 months that met three complementary feeding indicator requirements, including MMF, which was 41% (23). Ensuring adequate meal frequency poses a challenge in numerous developing countries, particularly in regions with low household food security, such as Sub-Saharan Africa.

The first 1,000 days of life are crucial for rapid growth, neurodevelopment, high nutritional needs, heightened sensitivity to programming effects, and increased vulnerability (24). Ensuring children’s nutritional wellbeing and improving their overall health is cost-effective through increasing meal frequency and diversity during the critical window period (25). Regularly meeting MMF can help prevent wasting (acute malnutrition) and reduce the risk of stunting (chronic malnutrition). For example, a study found that the prevalence of stunting was higher in children aged 6–8 months, highlighting the impact of inadequate complementary feeding on child growth (26). MMF, a key risk factor for stunting, is an easily measurable indicator that provides immediate feedback about feeding practices, enabling prompt, actionable responses such as education, behavior change programs, and policy interventions to prevent malnutrition.

In SSA, efforts have been made to decrease undernutrition and enhance IYCF practices. The Accelerating Nutrition Improvements (ANI) scale-up initiative was implemented in partnership with the Ministry of Health and WHO in Ethiopia, Uganda, and Tanzania (27). Interventions involving behavior change strategies significantly improved maternal and child nutrition outcomes by educating caregivers about optimal IYCF practices (28). However, SSA showed a prevalence level below half for MMF, with Western and Central Africa at 39.6% and Eastern and Southern Africa at 45.1% (29). Studies have been conducted on the factors associated with MMF in different countries across SSA (5, 14, 30–32). Nevertheless, these studies had limitations on generalizability to the regional context. The present study sought to address this gap and make a generalizable regional comparison using the most recent demographic and health survey (DHS) data in SSA. This study aims to contribute to the global debate on MMF by providing a detailed analysis of the magnitude and contributing factors. Our study not only identified the key individual and contextual determinants but also offered recommendations for policymakers. By filling the gaps, the study expected to increase understanding of how to improve minimum recommended meal frequency practices, thereby supporting global efforts to reduce malnutrition and promote better health outcomes for children.

## Methods

### Study design, setting, and period

We used data from the latest standard demographic and health survey in SSA countries carried out since 2010 to obtain up-to-date information. The Demographic and Health Surveys (DHS) are nationally representative household surveys that gather data on a wide range of population, health, and nutrition indicators. These surveys are conducted every 5 years to provide current information and enable comparisons over time. The DHS surveys cover various topics, including child health and nutrition. We utilized DHS data from all available Sub-Saharan African countries to comprehensively understand regional health and make cross-country comparisons (33). In this study, we included a total of 35 countries from the four regions in the SSA: Eastern Africa, Central Africa, Western Africa, and Southern Africa (Table 1) (34). Every survey is carried out at the national level and represents its respective country. This makes it a large-scale study with significant sample sizes. The participants in all these surveys were chosen using a two-stage cluster sampling method (35).

**Table 1:** Sample size determination of MMF and associated factors among children aged 6–23 months in each SSA.

Region	Country	Minimum meal frequency		Weighted frequency
		Yes (%)	No (%)	
East Africa	Burundi	1,505 (37.2)	2,543 (62.8)	4,048
	Ethiopia	809 (54.2)	683 (45.8)	1,492
	Kenya	1,698 (35.1)	3,134 (64.9)	4,832
	Comoros	264 (29.7)	624 (70.3)	888
	Madagascar	2,237 (64.0)	1,260 (36.0)	3,497
	Malawi	1,349 (28.7)	3,361 (71.3)	4,710
	Mozambique	1,402 (41.0)	2,020 (59.0)	3,422
	Rwanda	1,019 (43.9)	1,301 (56.1)	2,320
	Tanzania	964 (31.6)	2,085 (68.4)	3,049
	Uganda	1,619 (40.3)	2,401 (59.7)	4,020
	Zambia	1,081 (40.6)	1,585 (59.4)	2,666
	Zimbabwe	564 (34.4)	1,075 (65.6)	1,639
Central Africa	Angola	1,173 (31.4)	2,560 (68.6)	3,733
	Congo democratic republic	1,672 (34.6)	3,164 (65.4)	4,836
	Congo	543 (23.6)	1,762 (76.4)	2,305
	Cameroon	1,108 (42.5)	1,497 (57.5)	2,605
	Gabon	867 (50.3)	856 (49.7)	1,723
	Chad	1,678 (37.3)	2,816 (62.7)	4,493
West Africa	Burkina Faso	1,179 (31.4)	2,069 (68.6)	3,248
	Benin	1,668 (43.3)	2,182 (56.7)	3,850
	Cote d’Ivoire	684 (26.2)	1,932 (73.8)	2,616
	Ghana	694 (43.1)	915 (56.9)	1,609
	Gambia	1,013 (49.9)	1,017 (50.1)	2,030
	Guinea	452 (23.9)	1,435 (70.1)	1,887
	Liberia	292 (21.4)	1,072 (78.6)	1,364
	Mali	838 (28.8)	2,069 (71.2)	2,907
	Mauritania	773 (25.0)	2,315 (75.0)	3,088
	Nigeria	3,668 (40.5)	5,392 (59.5)	9,060
	Niger	1,745 (51.0)	1,673 (49.0)	3,418
	Sierra Leone	810 (30.9)	1,812 (69.1)	2,622
	Senegal	552 (33.8)	1,081 (66.2)	1,634
	Togo	907 (45.8)	1,074 (54.2)	1,981
Southern Africa	Lesotho	521 (59.8)	351 (40.2)	872
	Namibia	494 (40.8)	717 (59.2)	1,212
	South Africa	437 (51.5)	411 (48.5)	848

### Population and eligibility criteria

The source population is all children between the ages of 6 and 23 months in the 5 years preceding the survey period, encompassing the 35 SSA countries. The study population included children aged 6–23 months in the selected enumeration areas. For mothers with more than one child, only the youngest child was included. Furthermore, only children who lived with their mothers or caretakers were included. Ultimately, 101,558 children were included (100,526 after weighting). Most of the participants were from Nigeria, with 9,060 children, whereas South Africa had the lowest number of children, with 848 when weighted (Table 1).

### Sampling procedure

In total, 43 SSA countries have DHS reports. However, data were unavailable for Cape Verde and Equatorial Guinea. Central Africa Republic, Eswatini, Sao Tome and Principe, and Sudan were excluded as they had no DHS reports after 2010. Botswana and Eritrea were also excluded due to the restriction of data. As a result, 35 SSA countries were included in the study, and the analysis was conducted using the kids’ record (KR) dataset.

### Study variables

#### Outcome variables

The dependent variable was minimum meal frequency (MMF), which is defined as the proportion of breastfed and non-breastfed children aged 6–23 months who receive solid, semisolid, or soft foods (but also including milk feeds for non-breastfed children) the minimum number of times or more (36).

The minimum number of times is defined as follows:

- 2 times for breastfed infants aged 6–8 months,
- 3 times for breastfed children aged 9–23 months, and.
- 4 times for non-breastfed children 6–23 months (8).

If the child achieved the MMF, it was coded as “yes = 1,” whereas if the child did not achieve the MMF, it was coded as “no = 0.”

#### Explanatory variables

Both individual-and community-level independent variables have been studied. The individual-level factors included were maternal age, head of the household, working status of the mother, educational level of the mother, marital status, wealth index, media exposure, antenatal care follow-up, mode of delivery, birth order, preceding birth interval, sex of the child, multiple births, age of the child, and current breastfeeding status. Media exposure was created through watching TV and listening to radio. The community-level variables included region in SSA, residence, and income level. The income level was categorized as lower income, lower-middle income, or upper-middle income according to the World Bank list of economic classifications (37).

### Data processing and analysis

Stata (StataCorp, USA) version 17.0 was used to perform all the statistical analyses. First, each SSA country was given a code and appended together to create a single dataset. Then, both the dependent and independent variables were extracted, cleaned, coded, and analyzed. We used weighted data to ensure the survey’s representativeness.

#### Model building

By considering the hierarchical nature of DHS data, a multilevel Poisson regression model was fitted to determine the impact of each independent variable on the MMF. DHS was a cross-sectional study, and the prevalence of MMF among children aged 6–23 months in SSA was 38.47%, which was greater than 10%. In this case, reporting the odds ratio from binary logistic regression could overestimate the relationship between the dependent and independent variables. As a result, the prevalence ratio serves as the most appropriate measure of association for this study. We used a multilevel Poisson regression model with robust variance to assess factors associated with MMF (38, 39). In addition, the multilevel robust Poisson regression model surpassed the multilevel log-binomial regression model regarding convergence.

Bi-variable multilevel Poisson regression analysis was conducted to identify variables suitable for inclusion in the multivariable analysis. Variables that had a *p* < 0.2 in this analysis and those that were deemed significant based on literature were considered potential candidates for the multivariable analysis. Four models were fitted for the multivariable multilevel Poisson regression analysis. The first was the null model, which was fitted to check the variability of the MMF across the cluster, and it contains no independent variables. The second model (Model 2) contains individual-level variables only. The third (Model 3) contains community-level variables. In the last model (Model 4), both individual-and community-level variables were fitted simultaneously with the prevalence of MMF. The above models were compared via the deviance and log-likelihood test, and the model with the highest log-likelihood ratio and the lowest deviance was selected as the best-fit model. In addition, the variance inflation factor (VIF) was used to detect multicollinearity, and all variables had VIF values less than 10, with a mean VIF of 1.72 (Supplementary Table 1).

#### Parameter estimation method

Fixed effects are measures of association and were used to estimate the relationship between the prevalence of MMF and the independent variables at the individual and community levels. The associations are presented as adjusted prevalence ratios (aPR) and 95% confidence intervals (CI), and variables with a *p* < 0.05 in the multivariable analysis were considered as significant predictors of MMF among children aged 6–23 months in SSA. The random effects or the measures of variation were estimated by the median odds ratio (MOR) and intraclass correlation coefficient (ICC). The ICC was estimated to assess the clustering effect, and the MOR was estimated to quantify the variation or heterogeneity in the MMF between clusters in terms of the odds ratio scale (40, 41).

### Ethics approval and consent to participate

The data were obtained from the Demographic and Health Surveys Program with no personal identifiers and can be freely accessed from the program website.^1^ As the study was a secondary data analysis of publicly available data from the MEASURE DHS program and there was no interaction between the researcher and the participants, ethical approval and participant consent were not necessary for this particular study.

## Results

### Background characteristics of the study population

A weighted total of 100,526 children aged 6–23 months 5 years preceding the survey in SSA countries were included in this study. Of these, 41,315 (41.1%) respondents were from the western region of Africa, and 2,931 (2.9%) respondents were from the southern region. The percentage of MMF in the southern region of Africa (49.5%) was greater than that in the other regions. A total of 67,165 (66.8%) respondents were rural residents, and the proportion of MMF was greater among urban children. More than half (58.9%) of the countries have lower income levels, while the upper-middle-income countries have a higher proportion of MMF (Table 2).

**Table 2:** Characteristics of mothers and children by the prevalence of MMF in SSA.

Variable		Minimum meal frequency		Weighted frequency (%)
		Yes (%)	No (%)	
Age of the mother	15–19	3,493 (36.8)	5,998 (63.2)	9,491 (9.4)
	20–35	28,756 (38.1)	46,663 (61.9)	75,419 (75.0)
	36–49	6,030 (38.6)	9,585 (61.4)	15,615 (15.5)
Educational level of the mother	No education	12,612 (35.0)	23,409 (65.0)	36,021 (35.8)
	Primary	12,286 (37.0)	20,948 (63.0)	33,234 (33.1)
	Secondary and above	13,381 (42.8)	17,890 (57.2)	31,271 (31.1)
Working status of the mother	Not working	14,132 (36.1)	25,057 (63.9)	39,189 (39.6)
	Working	23,317 (39.0)	36,453 (61.0)	59,770 (60.4)
Marital status	Single	11,259 (36.0)	20,051 (64.0)	31,310 (31.2)
	Married	27,021 (39.0)	42,195 (61.0)	69,216 (68.8)
Family size	1–6	22,255 (38.1)	36,097 (61.9)	58,352 (58.1)
	7–10	11,217 (37.9)	18,371 (62.1)	29,588 (29.4)
	Above 10	4,807 (38.2)	7,779 (61.8)	12,586 (12.5)
Wealth index	Poor	15,332 (35.5)	29,042 (65.5)	44,374 (44.2)
	Middle	7,652 (37.8)	12,574 (62.2)	20,226 (20.1)
	Rich	15,295 (42.6)	20,631 (57.4)	35,926 (35.7)
Media exposure	No	14,251 (34.9)	26,625 (65.1)	40,876 (40.7)
	Yes	24,028 (40.3)	35,618 (59.7)	59,646 (59.3)
Sex of household head	Male	30,830 (38.5)	49,210 (61.5)	80,040 (79.6)
	Female	7,449 (36.4)	13,036 (63.6)	20,485 (20.4)
Age of the child in months	6–11	13,671 (38.9)	21,472 (61.1)	35,143 (35.0)
	12–17	13,434 (37.9)	22,008 (62.1)	35,442 (35.2)
	18–23	11,174 (37.3)	18,767 (62.7)	29,941 (29.8)
Antenatal care visit	No	3,971 (35.3)	7,269 (64.7)	11,240 (11.3)
	1–3 times	11,614 (37.3)	19,533 (62.7)	31,147 (31.3)
	4 or more times	22,644 (39.6)	34,578 (60.4)	57,222 (57.4)
Delivery by cesarean section	No	35,508 (37.7)	58,585 (62.3)	94,093 (93.8)
	Yes	2,692 (43.2)	3,544 (56.8)	6,236 (6.2)
Sex of the child	Male	19,338 (38.0)	31,599 (62.0)	50,937 (50.7)
	Female	18,941 (38.2)	30,648 (61.8)	49,589 (49.3)
Plurality	Single birth	37,636 (38.1)	61,191 (61.9)	98,827 (98.3)
	Multiple birth	644 (37.9)	1,055 (62.1)	1,699 (1.7)
Birth order	≤ 3	22,569 (38.7)	35,714 (61.3)	58,283 (58.0)
	Above 3	15,710 (37.2)	26,533 (62.8)	42,243 (42.0)
Preceding birth interval in months	No	8,741 (39.0)	13,668 (61.0)	22,409 (22.3)
	< 24	4,623 (36.5)	8,035 (63.5)	12,658 (12.6)
	24 and above	24,915 (38.1)	40,544 (61.9)	65,459 (65.1)
Birth weight	Large	12,687 (38.7)	20,139 (61.3)	32,826 (33.9)
	Average	18,553 (39.8)	28,042 (60.2)	46,595 (48.2)
	Small	5,743 (36.7)	9,895 (63.3)	15,638 (16.2)
	Unknown	477 (29.5)	1,138 (70.5)	1,615 (1.7)
Postnatal check-up within 2 months	No	22,536 (36.1)	39,863 (63.9)	62,399 (65.0)
	Yes	14,235 (42.3)	19,410 (57.7)	33,645 (35.0)
Currently breastfeeding	No	7,218 (34.6)	13,641 (65.4)	20,859 (20.8)
	Yes	31,058 (39.0)	48,598 (61.0)	79,656 (79.2)
Residence	Urban	13,391 (40.1)	19,970 (59.9)	33,361 (33.2)
	Rural	24,888 (37.1)	42,277 (62.9)	67,165 (66.8)
Region in SSA	Central Africa	7,041 (35.7)	12,655 (64.3)	19,696 (19.6)
	East Africa	14,512 (39.7)	22,071 (60.3)	36,583 (36.4)
	Southern Africa	1,452 (49.5)	1,479 (50.5)	2,931 (2.9)
	West Africa	15,274 (37.0)	26,041 (63.0)	41,315 (41.1)
Country income level	Lower	23,157 (39.1)	36,039 (60.9)	59,196 (58.9)
	Lower-middle	13,325 (35.5)	24,224 (64.5)	37,549 (37.3)
	Upper-Middle	1,798 (47.5)	1,984 (52.5)	3,782 (3.8)

### Maternal and child characteristics of the study population

The mean age of the mothers was 28 ± 6.8 years, and the average age of the mothers at their first birth was 19.4 ± 3.8 years. Among the mothers, 36,021 (35.8%) had no formal education, yet a larger proportion of MMF was observed among those who had attended secondary education or above. The majority of the mothers (68.8%) were married, 60.4% were not working, and 44,374 (44.2%) were of poor wealth status. The percentage of MMF was slightly greater among married women, working women, and those with rich wealth. Over half of the respondents (59.3%) had media exposure or listened to the radio or watched television. Over three-fourths (79.6%) of the participants’ household heads were males, and the proportion of MMF was greater among them. More than half of the women (57.4%) had four or more antenatal care visits (Table 2).

The children who were studied were 14.1 months old on average, with a standard deviation of ±5.1. More than half (50.7%) of the children aged 6–23 months were males, 35.2% were in the age group of 12–17 months, and 12.6% had a preceding birth interval of less than 2 years. Almost half of the respondents (48.2%) had average birth weights, and only 35% had postnatal check-ups within 2 months. Almost all the children were single births (98.3%) and were not delivered by cesarean section (93.8%) (Table 2).

### Pooled prevalence of MMF among children aged 6–23 months

**Figure 1:**
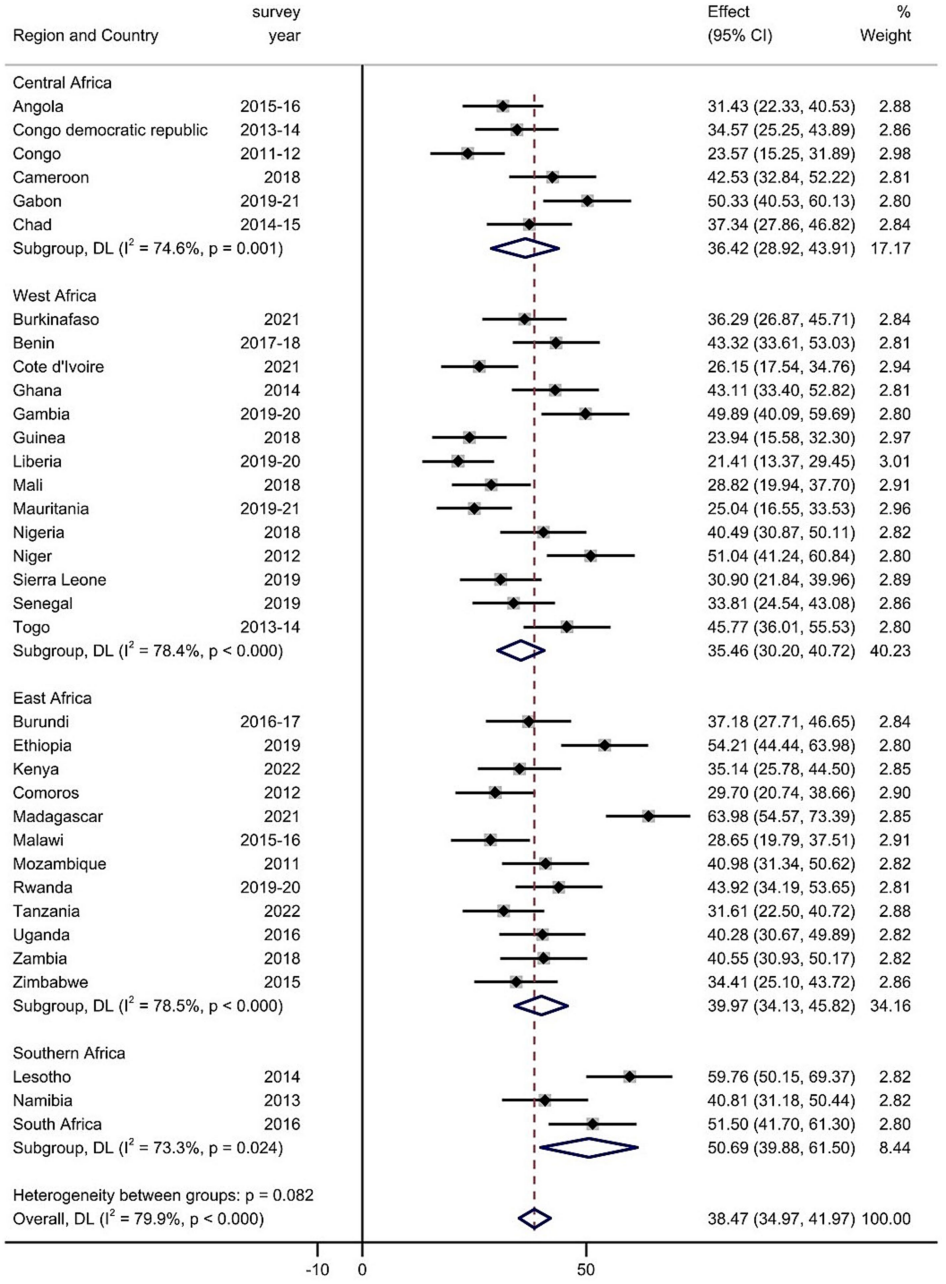
Forest plot of the pooled magnitude of MMF among children aged 6–23 months in SSA.

The prevalence of MMF differs across SSA countries. The prevalence of MMF was lowest in Liberia, at 21.41% (95% CI: 13.37–29.45), whereas the highest prevalence was recorded in Madagascar, at 63.98% (95% CI: 54.57–73.39%). The pooled prevalence of MMF among children aged 6–23 months in SSA was 38.47% (95% CI: 34.97–41.97) (Figure 1).

Subgroup analyses were performed on the basis of the region in SSA and the level of income of the country. The pooled magnitude of MMF intake ranged from 35.46% (95% CI: 30.20, 40.72%) in West Africa to 50.69% (95% CI: 39.88, 61.50%) in South Africa based on subgroup analysis of regions in SSA (Figure 1). Moreover, the pooled magnitude of MMF across SSA country income levels was determined. The pooled estimate of MMF in low-income countries was 39.01% (95% CI: 34.02, 43.99%), that in lower-middle-income countries was 35.64% (95% CI: 30.36, 40.92%), and that across upper-middle-income countries was 47.49% (95% CI: 40.82, 54.16%) (Supplementary Figure 1).

### Determinants of MMF among children aged 6–23 months in SSA

Random effect: The ICC and MOR were assessed. The ICC in the null model was 0.186, suggesting a clustering effect. This implies approximately 19% of the variation in MMF among children aged 6–23 months was attributed to cluster differences, whereas the remaining 81% was attributed to individual-level factors. The MOR value of 2.29 in the null model also revealed that the odds of MMF among the study subjects were different between the clusters. If we randomly select children from two different clusters, those from a cluster with a higher MMF would have 2.29 times the odds of having MMF compared with those from a cluster with a lower MMF. Model 4 was chosen as the best-fitted model as it was the model with the lowest deviance (121365.48).

Fixed effect: According to the results of the final model (Model 4), marital status, sex of household head, education level, working status, wealth index, media exposure, age of the child, postnatal check-up, current breastfeeding, residence, and region in SSA were significantly associated with the dependent variable MMF among children aged 6–23 months.

Infants and young children whose mothers are married had 1.07 times (aPR = 1.07; 95% CI: 1.04–1.09) higher prevalence of having MMF compared to those whose mothers are unmarried. Children whose mothers had secondary education and above had 1.15 times (aPR = 1.15; 95% CI: 1.12, 1.18) higher prevalence of having MMF than those whose mothers did not have formal education. The prevalence of MMF among children of female household heads decreased by 5% (aPR = 0.95; 95% CI: 0.93, 0.97) compared to children of male household heads. The prevalence of receiving MMF was 1.10 times higher among children whose mothers were working (aPR = 1.10; 95% CI: 1.08, 1.12), compared to their counterparts. Children from middle and rich households had 7 and 18% higher prevalence of MMF than those from poor households (aPR =1.07; 95% CI: 1.04, 1.09) and (aPR =1.18; 95% CI: 1.15, 1.21), respectively. Compared with children whose mothers had no media exposure, those whose mothers had media exposure had an increased prevalence of receiving MMF by 6% (aPR =1.06; 95% CI; 1.04, 1.08).

Children aged 18 to 23 months had 1.02 times (aPR =1.02; 95% CI: 1.01, 1.05) higher prevalence of receiving MMF than children aged 6 to 11 months. Compared with that of their counterparts, the prevalence of receiving MMF was 16% (aPR =1.16; 95% CI: 1.13, 1.18) higher among children who received postnatal check-ups within 2 months after delivery. Breastfeeding children had 16% (aPR =1.16; 95% CI: 1.13, 1.19) higher prevalence of MMF than non-breastfeeding children. Children who reside in rural areas were found to had a 3% (aPR = 1.03; CI: 1.01, 1.06) increased prevalence of receiving MMF than urban residents. We also found that children from Eastern, Southern, and Western Africa had 1.10 (aPR = 1.10; 95% CI: 1.06, 1.14), 1.38 (aPR = 1.38; 95% CI: 1.31, 1.46), and 1.04 (aPR = 1.04; 95% CI: 1.01, 1.07) times higher prevalence of receiving MMF than children from Central Africa (Table 3).

**Table 3:** Multivariable multilevel binary logistic regression analysis of individual-and community-level factors associated with MMF in SSA.

Variable	Null model	Model 2	Model 3	Model 4
		aPR (95% CI)	aPR (95% CI)	aPR (95% CI)
Age of the mother				
15–19		1		1
20–35		1.01 (0.98, 1.04)		0.99 (0.97, 1.03)
36–49		1.03 (0.97, 1.07)		1.01 (0.97, 1.05)
Educational level of the mother				
No education		1		1
Primary		1.05 (1.02, 1.07)		1.02 (0.99, 1.04)
Secondary and above		1.17 (1.14, 1.20)		1.15 (1.12, 1.18)***
Working status of the mother				
Not working		1		1
Working		1.09 (1.07, 1.11)		1.10 (1.08, 1.12)***
Marital status				
Single		1		1
Married		1.07 (1.04, 1.09)		1.07 (1.04, 1.09)***
Wealth index				
Poor		1		1
Middle		1.06 (1.03, 1.08)		1.07 (1.04, 1.09)***
Rich		1.16 (1.13, 1.19)		1.18 (1.15, 1.21)***
Media exposure				
No		1		1
Yes		1.05 (1.03, 1.08)		1.06 (1.04, 1.08)***
Sex of household head				
Male		1		1
Female		0.96 (0.94, 0.98)		0.95 (0.93, 0.97)**
Age of the child in month				
6–11		1		1
12–17		0.99 (0.97, 1.01)		0.99 (0.97, 1.01)
18–23		1.02 (1.01, 1.05)		1.02 (1.01, 1.05)*
Antenatal care visit				
No		1		1
1–3 times		1.01 (0.97, 1.04)		0.99 (0.96, 1.03)
4 or more times		1.01 (0.98, 1.04)		1.00 (0.97, 1.04)
Delivery by cesarean section				
No		1		1
Yes		1.04 (1.01, 1.07)		1.02 (0.99, 1.05)
Sex of the child				
Male		1		1
Female		1.01 (0.99, 1.02)		1.01 (0.99, 1.02)
Birth order				
≤ 3		1		1
Above 3		0.99 (0.97, 1.01)		0.99 (0.98, 1.02)
Preceding birth interval in months				
No		1		1
< 24		0.97 (0.94, 0.99)		0.98 (0.94, 1.01)
24 and above		0.99 (0.97, 1,01)		0.99 (0.97, 1.02)
Postnatal check-up within 2 months				
No		1		1
Yes		1.17 (1.45, 1.19)		1.16 (1.13, 1.18)***
Currently breastfeeding				
No		1		1
Yes		1.16 (1.13, 1.19)		1.16 (1.13, 1.19)***
Residence				
Urban			1	1
Rural			0.90 (0.88, 0.92)	1.03 (1.01, 1.06)*
Region in SSA				
Central Africa			1	1
East Africa			1.20 (1.16, 1.24)	1.10 (1.06, 1.14)***
Southern Africa			1.47 (1.40, 1.54)	1.38 (1.31, 1.46)***
West Africa			1.09 (1.06, 1.13)	1.04 (1.01, 1.07)*
ICC (%)	18.6 (17.8, 19.5)			
MOR	2.29			
Log-Likelihood	−75306.23	−70342.35	−75153.62	−70274.72
Deviance	150612.46	140684.7	150307.24	140549.44

aPR, adjusted prevalence ratio, *p < 0.05, **p < 0.01, and ***p∼0.

## Discussion

This study examines minimum meal frequency and its determinants among children aged 6–23 months in Sub-Saharan African countries. It revealed that the pooled prevalence of MMF in SSA was 38.47% (95% CI: 34.97–41.97). This is consistent with studies from SSA among 32 countries between 2010 and 2016 (41.9%) (42), SSA among 32 countries between 2010 and 2020 (41.2%) (43), and India (41.5%) (44). This finding is lower than those of studies conducted globally (52.2%) (22), in 80 low-and middle-income countries (56.2%) (29), China (75.1%) (45), and Pakistan (84.7%) (46). This discrepancy might be due to the limited access to nutritious food in SSA as it is challenging in terms of food security. In addition, poverty, economic constraints, and conflict in the region make it difficult for families to provide regular meals for their children.

The factors that were significantly linked to MMF were marital status, sex of the household head, education level, working status, wealth index, media exposure, age of the child, postnatal check-up, current breastfeeding, residence, and region in SSA. Children aged 6 to 23 months whose mothers were married were more likely to receive MMF, which is consistent with the findings of a study from Benin (47). This could be attributed to the fact that married mothers usually get support from their spouse, family, or community, resulting in adequate practices regarding feeding their children (48). Compared with children of male household heads, children of female household heads were less likely to receive MMF, which is consistent with the findings from Gambia (31). This might be because if a woman is the head of the household, she will bear the majority of the family’s responsibilities. Consequently, she may need to spend considerable time away from home to fulfill the family’s needs or work. As a result of these factors, along with other underlying circumstances, the chance of her spending time outside the home is high, which may lead to her inability to provide the recommended MMF for her child (31).

Compared with children whose mothers had no formal education, those whose mothers had secondary education and above had an increased prevalence of receiving MMF. This finding is congruent with the findings from previous studies in Ghana (5), Ethiopia (10), SSA (43), Gambia (31), Indonesia (49), and Bangladesh (50). A possible explanation is that uneducated mothers are unable to read health education materials provided while visiting health facilities, whereas educated mothers might read leaflets, magazines, or books, which increases their chance of obtaining nutritional education, as evidenced in a study based in Nepal (51). Furthermore, they are more flexible in their ability to acquire new knowledge and modify their behavior quickly with respect to healthy child feeding practices.

Our findings also revealed that the working status of the mother and the household wealth index are significant determinants of MMF among infants and children in SSA. Children of working mothers and middle or rich households were more likely to receive MMF than children of non-working mothers and poor households, respectively. This finding parallels the postulations of previous studies (30, 31, 49, 50, 52, 53). This could be due to the fact that working mothers have their own income and can provide for their children whether they obtain financial support from their partners or not (5). Compared with poor households, rich and middle households do not have a problem with food availability, as they are food secure, which increases the likelihood of giving food to children (30, 52). Children of mothers who were exposed to media were more likely to receive MMF than those whose mothers had no media exposure, which is supported by previous studies (50, 52, 54). Messages obtained from media are more easily adopted and enhance proper feeding practices as they are considered credible sources of health and nutritional information (55).

In this study, we found that children aged 18–23 months were more likely to receive MMF than those aged 6–11 months. This observation is in accordance with the findings from previous research (5, 30, 31, 49). The possible justification might be that older children have the ability to ask for or obtain food from family members and even from the community and that the discontinuation or decreased frequency of breastfeeding as children grow older increases their chances of eating food. All these factors ensure that they meet the minimum requirement for a daily meal (5, 31). Children who received a postnatal check-up within 2 months after delivery were more likely to receive MMF than children with no postnatal check-up. This finding aligns with the results of previous studies (30, 52, 56, 57). This is because postnatal care clinics deliver information that increases knowledge about appropriate complementary feeding practices as part of the counseling service. Health professionals also advise mothers about the consequences of inappropriate child feeding practices as it can lead to morbidity and mortality (30, 58).

We found a statistically significant association between residence and MMF, in which the prevalence of MMF was higher among rural residents. Children from urban areas have better access to healthcare services, diverse food options, maternal education, and ideal family size. However, urban poverty and inequality made the urban advantage not distributed equally, and individuals living in urban poverty have a compromised right to food (59). The regions in Africa also play a role in determining the minimum meal frequency among infants and young children. Compared with living in the Central Africa region, living in Eastern and Southern Africa regions was associated with increased prevalence of receiving MMF among children. It was also revealed that children who were breastfeeding were more likely to receive MMF than their counterparts. This finding is in line with a previously conducted study (7). This might be because the frequency of meals for breastfeeding children is lower than that for non-breastfeeding children. To meet the requirements for MMF, non-breastfeeding children are advised to consume a minimum of four meals per day, whereas breastfeeding children have at least one fewer meal.

### Limitations of the study

This study is not without limitations; that is, owing to the cross-sectional nature of the data, the findings cannot establish a cause-and-effect relationship between minimum meal frequency and the explanatory variables. Furthermore, DHS surveys are based on self-reported information, which is likely to be prone to social desirability bias. Although the data were the most recent of these countries, the use of data from different time periods could have affected the comparability of the results.

### Areas for further research

Based on the findings and limitations of our study, there are areas of further research areas which have not been explored well. Examining the inequalities between children who receive the minimum adequate meal frequency and those who do not reveals potential disparities and the factors contributing to these inequalities. In addition, longitudinal studies are recommended to establish a cause-and-effect relationship between the dependent and independent variables and track changes in minimum meal frequency over time. Although factors such as maternal educational status have been examined, the impact of maternal knowledge and attitude regarding infant and young child feeding requires further exploration to better understand how these factors influence meal frequency. Finally, further research is needed to evaluate the impact of existing nutrition policies and programs on improving meal frequency across various countries in Sub-Saharan Africa.

## Conclusion and recommendations

The pooled prevalence of minimum meal frequency among children aged 6–23 months in Sub-Saharan Africa was 38.47%, which is low. This poses a significant challenge for preventing malnutrition and achieving health-and nutrition-related SDGs. To improve the MMF, infant and young child feeding policies should be strengthened by monitoring and evaluating these policies. The factors that were significantly associated with MMF were marital status, maternal education, sex of household head, maternal working status, wealth index, media exposure, child age, postnatal check-up, breastfeeding status, and SSA region. The study recommends promoting breastfeeding according to WHO guidelines and postnatal check-ups within 2 months after delivery. Children aged 6–11 months and those from low-income households should also be targeted to encourage feeding with MMF by providing support to families. Women should be empowered in various aspects of life, including social and economic spheres. Enhancing women’s education and financial independence through an increased number of educated, working mothers is crucial for improving the nutritional and health outcomes of both mothers and their children. The collective work of various implementing agencies such as governments, non-governmental organizations, and community groups is essential for the success and sustainability of these initiatives.

## Data Availability

Publicly available datasets were analyzed in this study. The raw dataset used and analyzed in this study can be accessed from the DHS website (https://dhsprogram.com/data/dataset_admin/index.cfm).

## Footnotes

^1^ www.dhsprogram.com
