# Supplementary material for: Minimum meal frequency and associated factors among children aged 6–23 months in Sub-Saharan Africa: a multilevel analysis of the demographic and health survey data
Source: Front Public Health. 2024 Nov 22;12:1468701. doi: 10.3389/fpubh.2024.1468701 (PMC11621055; doi:10.3389/fpubh.2024.1468701)
Supplement: Supplementary file 1 [file Image_1.pdf]

Minimum meal frequency and associated factors among children aged 6-23 months in Sub-Saharan Africa: A multilevel analysis of the demographic and health survey data

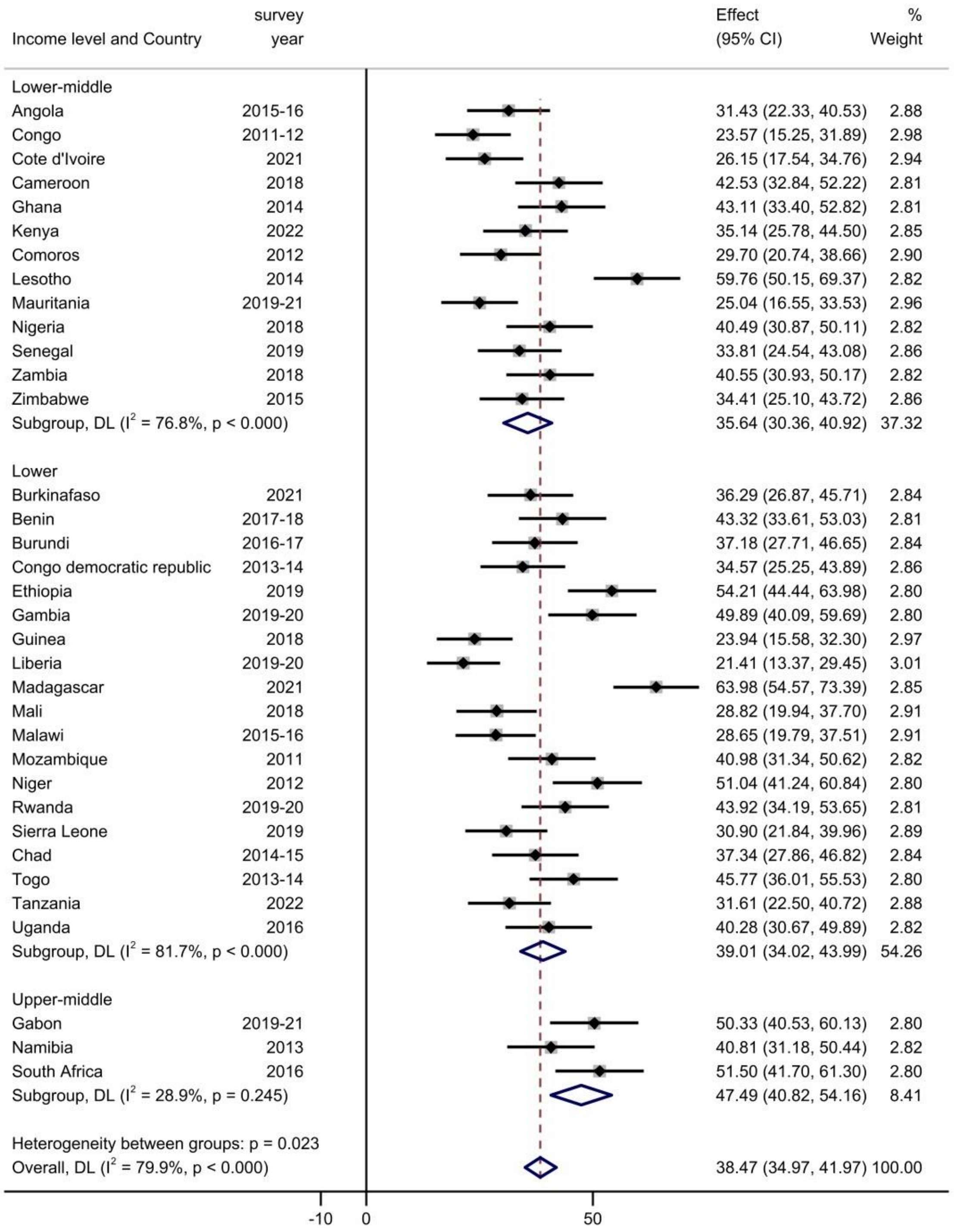

Supplementary Figure 1: Forest plot of pooled magnitude of MMF among children aged 6-23 months in SSA by country income level
