# Supplementary material for: Minimum meal frequency and associated factors among children aged 6–23 months in Sub-Saharan Africa: a multilevel analysis of the demographic and health survey data
Source: Front Public Health. 2024 Nov 22;12:1468701. doi: 10.3389/fpubh.2024.1468701 (PMC11621055; doi:10.3389/fpubh.2024.1468701)
Supplement: Supplementary file 2 [file Table_1.pdf]

Minimum meal frequency and associated factors among children aged 6-23 months in Sub-Saharan Africa: A multilevel analysis of the demographic and health survey data

Supplementary Table 1: Variance inflation factor result table

Variables		VIF	1/VIF
Age of the mother	15-19	ref	ref
	20-35	2.97	0.34
	36-49	3.31	0.30
Educational level of the mother	No education	ref	ref
	Primary	1.55	0.65
	Secondary and above	1.94	0.52
Working status of the mother	Not working	ref	ref
	Working	1.08	0.93
Marital status	Single	ref	ref
	Married	1.25	0.80
Wealth index	Poor	ref	ref
	Middle	1.26	0.79
	Rich	2.04	0.64
Media exposure	No	ref	ref
	Yes	1.34	0.75
Sex of household head	Male	ref	ref
	Female	1.10	0.91
Age of the child in month	6-11	ref	ref
	12-17	1.32	0.76
	18-23	1.54	0.65
Antenatal care visit	No	ref	ref
	1-3 times	2.69	0.37
	4 or more times	2.83	0.35
Delivery by cesarean section	No	ref	ref
	Yes	1.05	0.95
Sex of the child	Male	ref	ref
	Female	1.00	0.99
Birth order	≤ 3	ref	ref
	Above 3	1.55	0.65

Preceding birth interval in months	No	ref	ref
	< 24	1.73	0.58
	24 and above	2.17	0.46
Postnatal check-up within 2 months	No	ref	ref
	Yes	1.07	0.94
Currently breastfeeding	No	ref	ref
	Yes	1.27	0.79
Residence	Urban	ref	ref
	Rural	1.52	0.66
Region in SSA	Central Africa	ref	ref
	East Africa	2.02	0.49
	Southern Africa	1.69	0.59
	West Africa	2.09	0.48
Country income level	Lower	ref	ref
	Lower-middle	1.18	0.84
	Upper-Middle	1.96	0.51
**Mean VIF**		**1.72**	

vif – variance inflation factor**,** ref – reference category
